# Central Odontogenic Fibroma: A Rare Benign Tumor With Potentially Life‐Threatening Implications

**DOI:** 10.1002/ccr3.70761

**Published:** 2025-08-08

**Authors:** Farhad Ghorbani, Jamaledin Motazedian, Mohammad Saleh Khaghaninejad, Haleh Keshvari, Maryam Paknahad

**Affiliations:** ^1^ Oral and Maxillofacial Surgery Shiraz University of Medical Sciences Shiraz Iran; ^2^ Oral and Maxillofacial Pathologist Rajaei Hospital Shiraz Iran; ^3^ Oral and Dental Disease Research Center, Oral and Maxillofacial Radiology Department Shiraz Iran

**Keywords:** benign tumor, central odontogenic fibroma, mandible, slow‐growing

## Abstract

Central odontogenic fibroma (COF) is a rare benign tumor originating from periodontal tissues. Although it typically grows slowly and remains asymptomatic, COF can cause significant complications when neglected, including displacement of teeth, bone destruction, and even life‐threatening airway obstruction.

**Summary**

- Neglected COFs can lead to severe complications, including airway obstruction.
- Early diagnosis, timely surgical intervention, and interdisciplinary collaboration are crucial for preventing life‐threatening consequences.
- Regular radiological monitoring and long‐term follow‐up are essential to detect potential recurrence and optimize patient outcomes, ensuring effective management of this rare but significant tumor.

## 1 Introduction

Central odontogenic fibroma (COF) is a rare benign tumor originating from the periodontal tissues. These tumors account for less than 1.5% of all odontogenic neoplasms and primarily arise in the maxilla or mandible. Although they are typically asymptomatic and slow‐growing, COFs can become clinically significant when they reach a size that affects adjacent structures. Due to their infrequent occurrence, the biological behavior, recurrence patterns, and optimal management strategies for COFs remain incompletely understood, posing diagnostic and therapeutic challenges for clinicians [1, 2].

Histopathologically, COF is characterized by fibrous or fibromyxomatous proliferation interspersed with varying amounts of inactive odontogenic epithelium. These features often lead to misdiagnosis, as COF shares similarities with other lesions such as desmoplastic fibroma, odontogenic myxoma, or inflammatory masses. Thus, accurate diagnosis requires a careful correlation of clinical, radiographic, and histopathological findings [3, 4].

From a radiological perspective, COF can present as a unilocular or multilocular lesion with distinct or, occasionally, ill‐defined borders. In rare cases, it may exhibit a mixed radiolucent and radiopaque appearance, further complicating its differentiation from malignancies like osteosarcoma or fibrosarcoma. Such presentations emphasized the importance of biopsy and histopathological confirmation to establish a definitive diagnosis [5, 6].

Although COF is benign, its potential for aggressive growth and recurrence necessitates a comprehensive treatment plan. In particular, large, neglected lesions can compress critical structures, such as the airway, leading to potentially life‐threatening complications. Surgical excision remains the cornerstone of treatment; however, the reported recurrence rates range widely, from 3.3% to 50%, depending on the completeness of resection and the presence of specific histopathological features [5, 7].

This report presents a case of a 41‐year‐old male with a neglected mandibular COF that grew to a size capable of causing airway obstruction. This case highlights the importance of early diagnosis, timely intervention, and long‐term follow‐up in managing this rare yet clinically significant tumor. It also contributes to the limited body of literature on COF by detailing the diagnostic process, surgical management, and pathological findings while underscoring the critical role of interdisciplinary collaboration and vigilance among clinicians and pathologists in accurately diagnosing and effectively managing rare odontogenic tumors.

## 2 Case Examination

**FIGURE 1:**
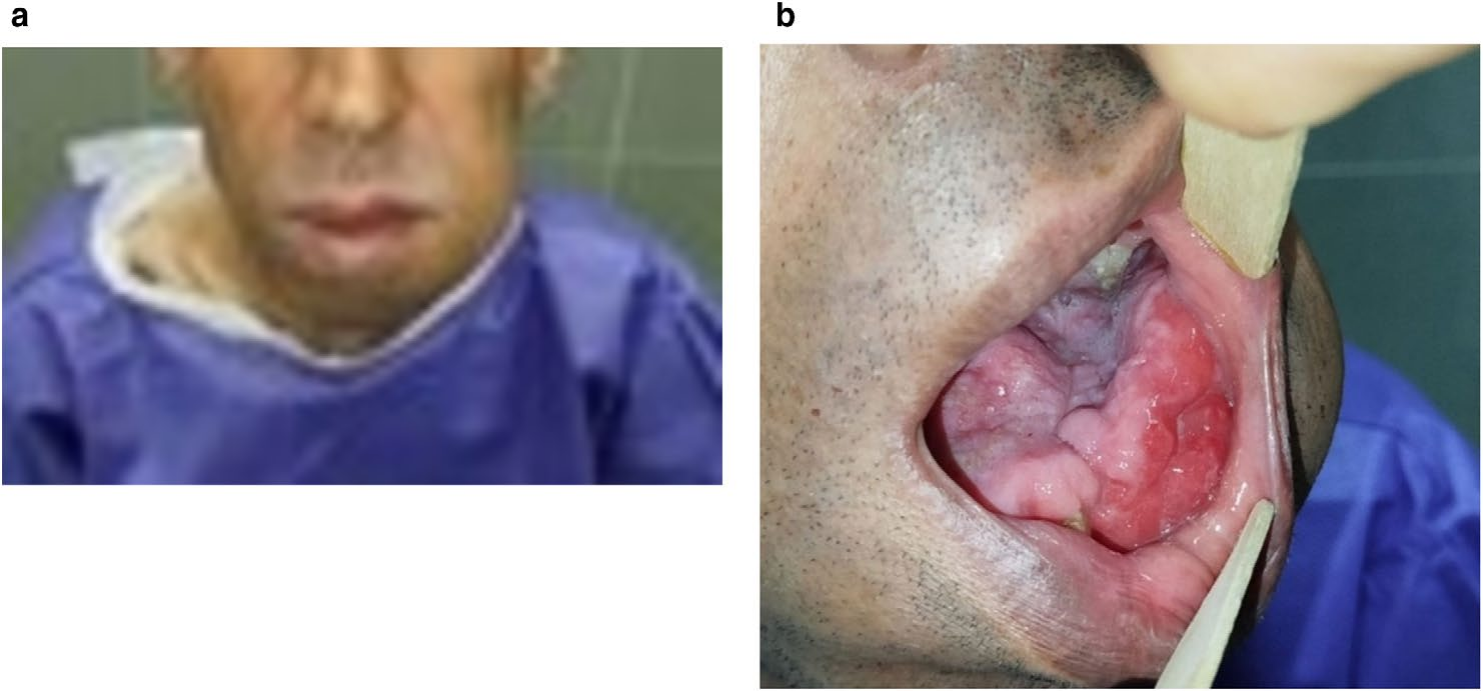
(a) The patient during general inspection with a swelling on the left mandible area. (b) Intraoral inspection showing an ill‐defined mass occupying the left side of the oral cavity and extending over the mandible midline and threatening the airway.

A 41‐year‐old male presented to the Oral and Maxillofacial Radiology Department with a complaint of a progressively enlarging mass in the left mandibular region, which he had noticed for the past 15 years. Written informed consent was obtained from the patient for the publication of this case report, including accompanying images. Initially, the lesion appeared as a small, painless lump that did not interfere with daily activities. However, over time, the patient experienced significant swelling and discomfort, which he ignored due to financial constraints and limited access to healthcare. The mass eventually caused noticeable facial asymmetry and functional impairments, including difficulty breathing and chewing (Figure 1a).

The patient's medical history was notable for two mandibular fractures from separate car accidents, which had forced him to use the left side of his mouth exclusively for mastication. This habit likely exacerbated the growth of the lesion due to increased mechanical stress on the affected area.

Extraoral examination revealed a large, firm, non‐tender swelling measuring approximately 5 × 7 cm on the left side of the mandible. The swelling extended from the left parasymphysis to the mandibular angle on the right side, causing marked facial asymmetry. The overlying skin showed no ulceration or discoloration.

Intraoral examination revealed diffuse swelling extending from the left mandibular second molar across the midline to the right premolar region. The lesion had displaced teeth and significantly narrowed the airway, resulting in breathing difficulties and sleep apnea. There were no signs of ulceration, bleeding, or infection in the oral cavity (Figure 1b).

## 3 Differential Diagnosis, Investigations, and Treatment

**FIGURE 2:**
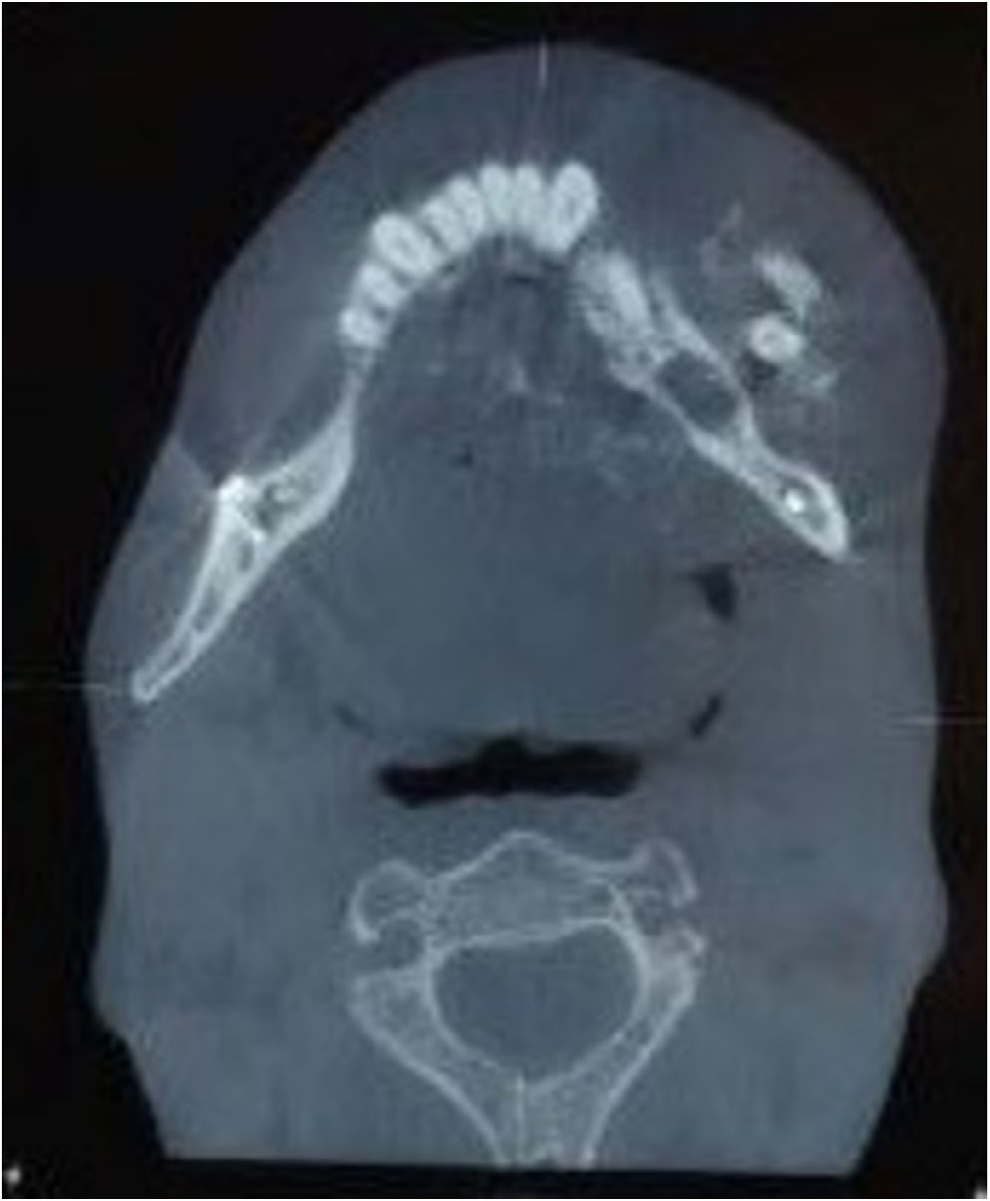
An ill‐defined destructive lesion in the left mandible that crosses the midline and extends to about the right premolar area with a sunburst appearance and coarse irregular new bone formation.

A head and neck computed tomography (CT) scan was performed to evaluate the lesion. The imaging revealed a poorly defined, mixed radiolucent and radiopaque lesion with a sunburst appearance and coarse, irregular new bone formation. The lesion extended from the left parasymphysis across the midline to the right mandibular premolar region, with evidence of cortical destruction (Figure 2). The differential diagnosis included osteosarcoma, fibrosarcoma, odontogenic myxoma, and central odontogenic fibroma.

**FIGURE 3:**
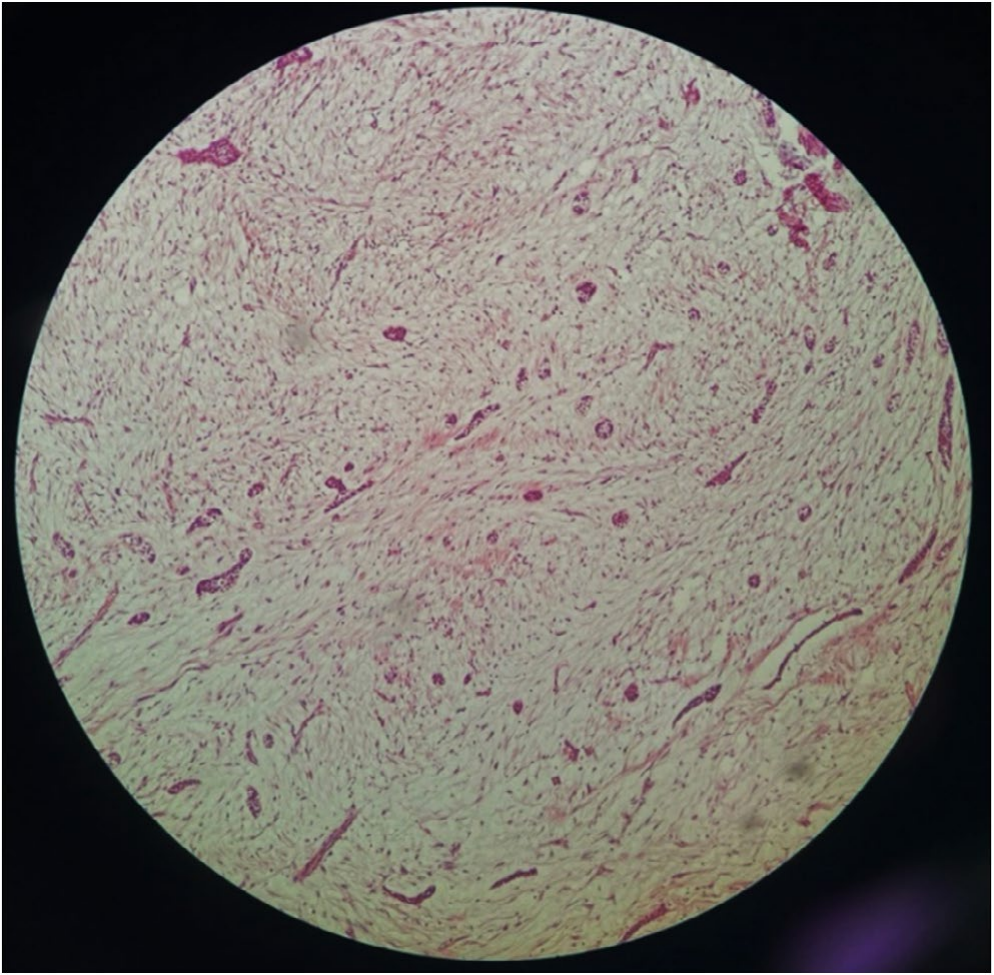
Microscopic examination shows odontogenic epithelium arranged as isolated nests or islands scattered in a loose fibrous connective tissue stroma.

An incisional biopsy was performed, and the specimen was sent for histopathological evaluation. The microscopic examination revealed inactive nests of odontogenic epithelium scattered within a loose fibrous stroma, consistent with a diagnosis of epithelium‐rich central odontogenic fibroma (COF). The absence of features such as atypia, mitotic activity, or necrosis supported the benign nature of the lesion (Figure 3).

**FIGURE 4:**
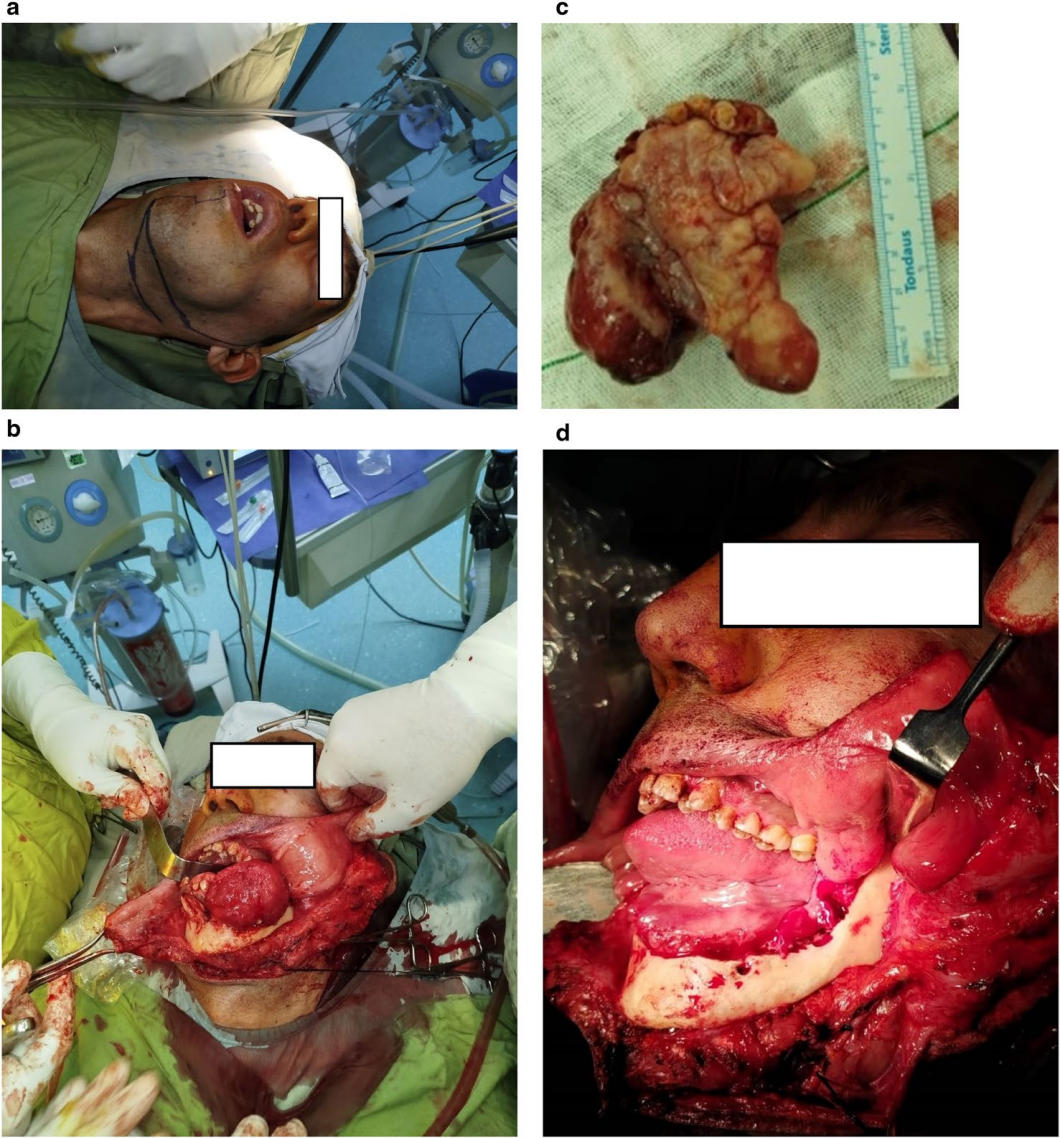
(a) Left submandibular lip‐split incision marking. (b) Myocutaneous flap reflection. (c) Resected mass after surgery. (d) The mandible resection during surgery.

Due to the severity of the airway obstruction, emergency surgery was planned. A tracheostomy was performed preoperatively to secure the airway. A left submandibular surgical excision was used to access the lesion, followed by the reflection of a myocutaneous flap for adequate exposure. Careful dissection preserved the left facial nerve and vascular structures. The lesion was completely excised via marginal resection, and selective lymph nodes from Zone I were sent for pathological evaluation, which revealed no malignancy (Figure 4a‑d).

## 4 Conclusion and Results (Outcome and Follow‐Up)

**FIGURE 5:**
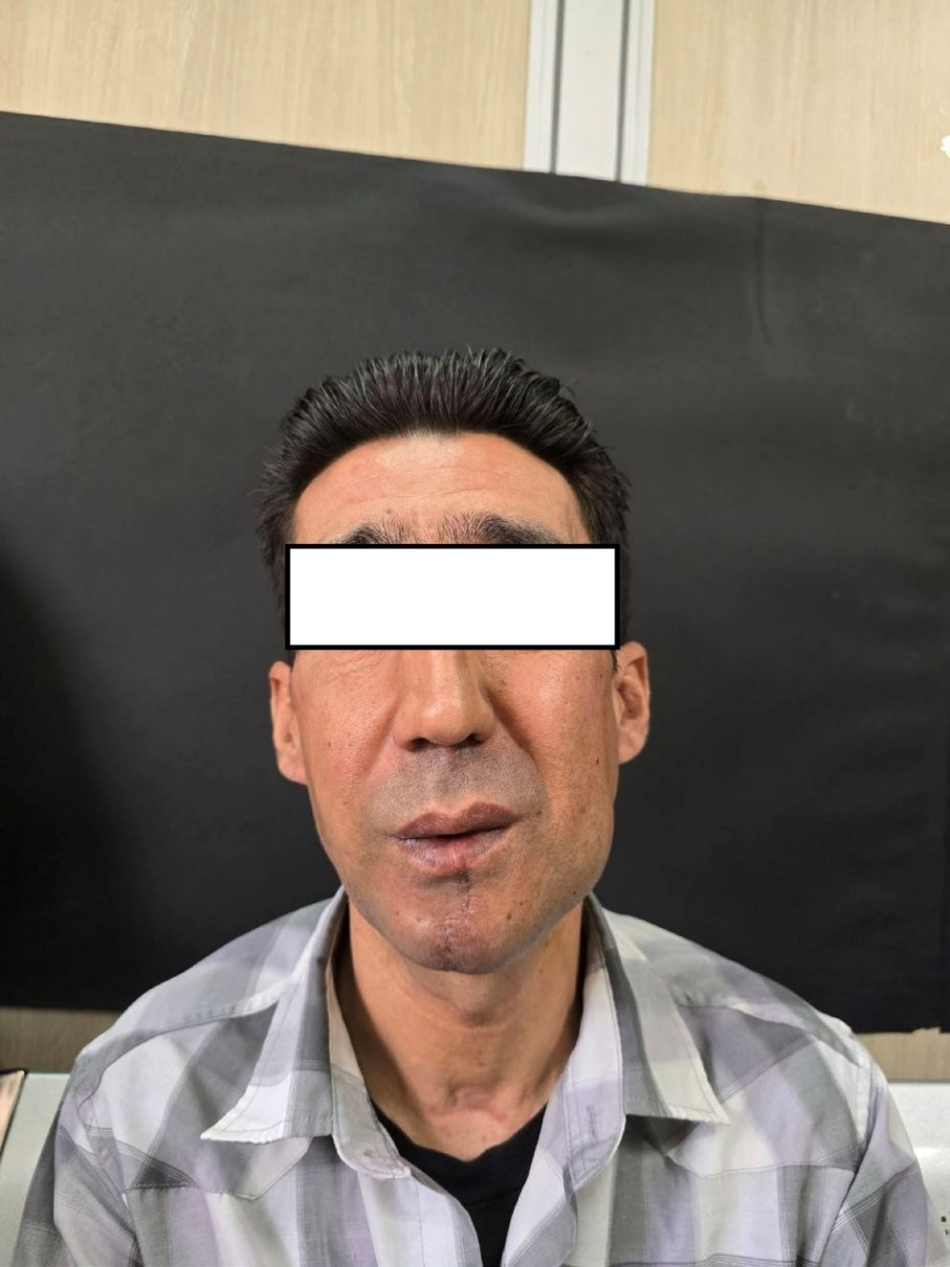
One‐year follow‐up.

Postoperative recovery was uneventful, with no signs of recurrence during a 3‐month follow‐up. A long‐term follow‐up plan involving biannual radiological assessments has been initiated to monitor for recurrence. Long‐term follow‐up with biannual imaging has been scheduled to monitor for potential recurrence (Figure 5).

## 5 Discussion

COF is an uncommon odontogenic tumor with unique clinical, radiological, and histopathological features. While typically benign and slow‐growing, this case illustrates the potential for COF to become life‐threatening when neglected, particularly due to its impact on critical structures like the airway. This discussion aims to contextualize the case within the broader literature, address the diagnostic and therapeutic challenges associated with COF, and highlight lessons for clinical practice.

The rarity of COF and its overlapping features with other odontogenic and non‐odontogenic lesions make diagnosis challenging. In this case, the lesion's clinical presentation—progressive mandibular swelling, displacement of teeth, and airway compromise—necessitated a thorough differential diagnosis. Radiological findings of a mixed radiolucent and radiopaque lesion with a “sunburst” appearance further complicated the initial assessment, as these features are more commonly associated with malignancies such as osteosarcoma or fibrosarcoma [8].

Histopathological evaluation remains the gold standard for confirming a COF diagnosis. The presence of inactive nests of odontogenic epithelium within a fibrous stroma is a hallmark feature. However, distinguishing COF from similar lesions such as desmoplastic fibroma or odontogenic myxoma requires careful correlation with clinical and radiological findings. For example, while odontogenic myxomas typically exhibit a more aggressive pattern of bone destruction, COFs often have a more localized and well‐defined presentation. These nuances emphasize the importance of multidisciplinary collaboration between surgeons, radiologists, and pathologists [9].

COFs are generally considered benign, but this case highlights their potential for aggressive growth under specific circumstances. In this patient, a history of mandibular fractures and unilateral mastication likely contributed to the lesion's rapid enlargement. This finding aligns with previous reports suggesting that mechanical stress and trauma can accelerate the growth of odontogenic tumors [10, 11].

Histologically, COFs are classified as epithelium‐rich or epithelium‐poor, depending on the extent of odontogenic epithelial content. Epithelium‐rich subtypes, as observed in this case, may carry a higher risk of recurrence due to their active biological behavior. Additionally, the presence of features such as budding basal cells and calcifications has been associated with higher recurrence rates. These observations highlight the need for comprehensive histopathological analysis to guide prognosis and follow‐up planning [12, 13].

Surgical excision remains the standard of care for COF. The approach may vary from enucleation to more extensive resections, depending on the size and extent of the lesion. In this case, the lesion's size and proximity to vital structures necessitated a tracheostomy for airway management and a surgical excision to ensure complete excision. Careful preservation of the facial nerve and adjacent vascular structures minimized postoperative complications and ensured functional recovery [2].

The unique challenges of untreated COF stem from its slow‐growing but progressively expanding nature, which can lead to severe functional and structural complications, particularly when the tumor is neglected for an extended period. While COF is classified as a benign odontogenic tumor, its unchecked growth can result in significant cortical expansion, displacement of adjacent structures, and, in extreme cases, life‐threatening airway compromise.

One of the most critical complications of untreated COF is its impact on the airway. As the lesion enlarges, it can cause progressive narrowing of the oropharyngeal and hypopharyngeal spaces, leading to difficulty in breathing, snoring, and eventually obstructive sleep apnea (OSA). In severe cases, as seen in this report, the compromised airway may necessitate emergency surgical intervention, such as tracheostomy, to secure ventilation. Unlike rapidly growing malignancies, COF's gradual but persistent growth can mask its severity, often leading to a delayed diagnosis until functional symptoms like dysphagia or dyspnea become evident.

Additionally, bone expansion and cortical thinning in untreated cases can increase the risk of pathological fractures, especially in patients with a history of trauma, as seen in this case. The lesion's ability to cross the midline and infiltrate surrounding anatomical structures adds to the complexity of surgical management, requiring a more extensive resection with potential postoperative morbidity. Ultimately, the challenges of neglected COF emphasize the critical need for early diagnosis, regular monitoring, and timely intervention to prevent severe complications, particularly those affecting airway patency and craniofacial stability.

Although recurrence is relatively uncommon, rates as high as 50% have been reported in some studies, particularly when excision is incomplete [7]. This variability underscores the importance of meticulous surgical technique and regular postoperative monitoring. Radiological follow‐up every 6 months, as planned in this case, is critical for early detection of recurrence.

While the overall prognosis for COF is excellent, the potential for recurrence and the challenges posed by large lesions underlines the need for long‐term follow‐up. This case showed how neglect and socioeconomic barriers can lead to advanced disease, emphasizing the role of patient education and healthcare accessibility in improving outcomes.

The diagnosis of COF is challenging due to its rarity and resemblance to other tumors. The slow‐growing and asymptomatic nature of COF often leads to delayed diagnosis. In this case, cortical expansion, midline crossing, and airway narrowing raised suspicion of a more aggressive pathology. The mixed radiolucent–radiopaque lesion with a sunburst appearance closely resembled osteosarcoma, while its well‐defined but expansile growth was similar to ossifying fibroma and odontogenic myxoma. The absence of rapid cortical destruction or periosteal reaction helped rule out malignancy. The presence of fibrous stroma with inactive odontogenic epithelium confirmed COF. The lack of atypia, mitotic activity, or necrosis distinguished it from fibrosarcoma and osteosarcoma, while the absence of ameloblastic differentiation ruled out ameloblastic fibroma. Accurate diagnosis required correlation of clinical, radiological, and histopathological findings, preventing misdiagnosis and overly aggressive treatment.

This case contributes valuable insights into the clinical behavior and management of COF. Key lessons include the following: the importance of early diagnosis and intervention to prevent complications from large lesions, the need for multidisciplinary collaboration in diagnosing and treating rare odontogenic tumors, and the significance of long‐term follow‐up to monitor for recurrence, particularly in epithelium‐rich subtypes. Additionally, this case demonstrates the need for further research into the biological mechanisms underlying COF's growth and recurrence. Developing standardized protocols for diagnosis, treatment, and follow‐up could improve patient outcomes and provide a clearer understanding of this rare tumor.

Future research on COF should focus on molecular and genetic studies to identify markers influencing pathogenesis and recurrence, longitudinal studies to understand growth patterns, and advanced imaging techniques (MRI, PET scans) to improve diagnostic accuracy. Investigating surgical vs. conservative treatment approaches will help optimize recurrence prevention while preserving functionality. Additionally, research on mechanical stress and trauma may reveal modifiable risk factors, while epidemiological studies can assess the impact of delayed diagnosis due to socioeconomic barriers. Overall, enhanced clinical, radiological, and molecular research is crucial to improving diagnostic precision, treatment strategies, and long‐term patient outcomes.

## 6 Conclusion

COF is a rare, benign tumor that can become potentially life‐threatening if neglected due to complications like airway compromise, as demonstrated in this case. Early diagnosis, multidisciplinary collaboration, and timely surgical intervention are crucial for managing such tumors. Despite its generally favorable prognosis, COFs require long‐term follow‐up due to the potential for recurrence, particularly in epithelium‐rich subtypes. This report highlights the importance of raising awareness about COF's clinical and pathological features and care to improve outcomes.

## Author Contributions

**Farhad Ghorbani:** validation, writing – review and editing. **Jamaledin Motazedian:** writing – review and editing. **Mohammad Saleh Khaghaninejad:** writing – original draft. **Haleh Keshvari:** writing – review and editing. **Maryam Paknahad:** writing – original draft.

## Consent

Written informed consent was obtained from the patient for the publication of this case report, including accompanying images.

## Conflicts of Interest

The authors declare no conflicts of interest.

## Data Availability

Data available on request from the authors.
